# 194. Progression of an Uncomplicated Urinary Tract Infection Among Female Patients with Susceptible and Non-Susceptible Urine Isolates: Findings from an Integrated Delivery Network

**DOI:** 10.1093/ofid/ofab466.194

**Published:** 2021-12-04

**Authors:** Jason Shafrin, Alen Marijam, Ashish V Joshi, Fanny S Mitrani-Gold, Katie Everson, Rifat Tuly, Peter Rosenquist, Michael Gillam, Maria Elena Ruiz

**Affiliations:** 1 PRECISIONxtract, Los Angeles, CA, USA; 2 GlaxoSmithKline plc., Collegeville, PA; 3 GlaxoSmithKline plc, Collegeville, PA; 4 PRECISIONheor, Austin, TX; 5 PRECISIONheor, Washington, DC; 6 MedStar Health, Washington, DC; 7 MedStar Washington Hospital Center, Washington, DC

## Abstract

**Background:**

Uncomplicated urinary tract infection (uUTI) is often treated empirically without antibiotic (AB) susceptibility testing; however, antimicrobial-resistant bacteria could lead to suboptimal treatment and progression to complicated UTI (cUTI). We examined the likelihood of uUTI progression to cUTI in patients with susceptible and non-susceptible uropathogens.

**Methods:**

We performed a retrospective cohort study using data from a large Mid-Atlantic US integrated delivery network’s electronic health records from July 1, 2016 to March 31, 2020. Patients included were female, aged ≥ 12 years with incident uUTI (diagnosis code or urine culture), and given an oral AB ± 5 days of diagnosis and ≥ 1 antibiotic susceptibility test. The primary outcome was progression to cUTI, defined as: new fever, nausea, or vomiting, in addition to uUTI symptoms; or receipt of intravenous antibiotic 3–28 days after index uUTI. Probability of progression to cUTI was assessed comparing patients with non-susceptible and susceptible isolates, with 1:1 propensity score matching. Patients retained for analysis had a nonzero predicted probability of being in the case and control group and were retained for analysis only if there were patients in the mirror group with similar propensity scores. Data were analyzed with logistic regression. Sensitivity analyses were performed to test the robustness of the primary analysis (**Table**).

**Results:**

A total of 2565 patients were included: 1030 (40.2%) had non-susceptible isolates and 1535 (59.8%) had susceptible isolates. Mean age was 43.5 years and 59.5% of the cohort was White. After propensity score matching, patients with non-susceptible isolates were more than twice as likely to progress to cUTI versus patients with sensitive isolates (10.7% versus 4.9%; odds ratio, 2.35; p < 0.001; **Figure**). In sensitivity analyses, patients with non-susceptible isolates remained significantly more likely to progress to cUTI (p ≤ 0.009), excluding those receiving fluoroquinolones only (**Table**).

Figure. Probability of progression to cUTI

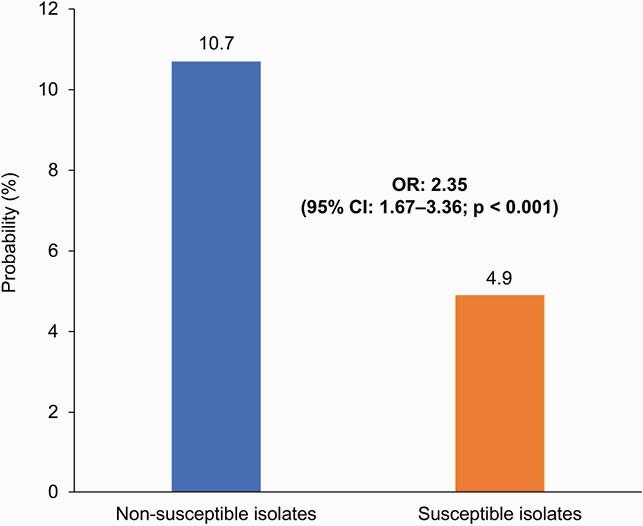

Table. Sensitivity analyses of the probability of uUTI progressing to cUTI in patients with non-susceptible versus susceptible isolates (matched population)

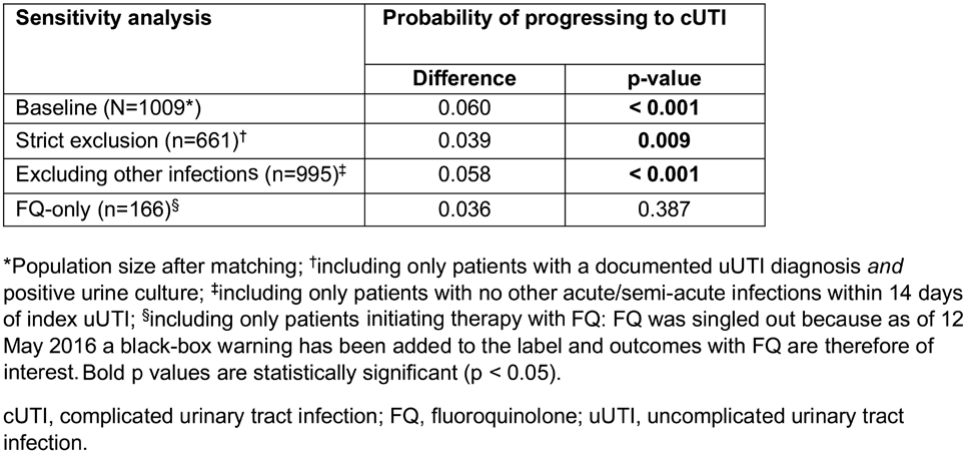

**Conclusion:**

Patients with uUTI and AB-resistant isolates were significantly more likely to progress to cUTI than those with susceptible isolates. This finding highlights the need for greater understanding of antimicrobial resistance and has implications for the clinical management of uUTI.

**Disclosures:**

**Jason Shafrin, PhD**, **Precision Medicine Group** (Employee, Former employee of Precision Medicine Group, which received funding from GlaxoSmithKline plc. to conduct this study) **Alen Marijam, MSc**, **GlaxoSmithKline plc.** (Employee, Shareholder) **Ashish V. Joshi, PhD**, **GlaxoSmithKline plc.** (Employee, Shareholder) **Fanny S. Mitrani-Gold, MPH**, **GlaxoSmithKline plc.** (Employee, Shareholder) **Katie Everson, MSc**, **Precision Medicine Group** (Employee, Employee of Precision Medicine Group, which received funding from GlaxoSmithKline plc. to conduct this study) **Rifat Tuly, MPH**, **Precision Medicine Group** (Employee, Employee of Precision Medicine Group, which received funding from GlaxoSmithKline plc. to conduct this study) **Peter Rosenquist, MSc**, **Precision Medicine Group** (Employee, Employee of Precision Medicine Group, which received funding from GlaxoSmithKline plc. to conduct this study) **Michael Gillam, MD**, **MedStar Health** (Employee, Employee of MedStar Health and received funding from GlaxoSmithKline plc. through Precision Medicine Group to conduct this study) **Maria Elena Ruiz, MD**, Nothing to disclose

